# Estimated Investment Need to Increase England's Capacity to Diagnose Eligibility for an Alzheimer's Treatment to G7 Average Capacity Levels

**DOI:** 10.14283/jpad.2024.24

**Published:** 2024-02-07

**Authors:** Soeren Mattke, Z. Shi, M. Hanson, S. Mitchell, C. Lynch, K. MacLean Kalonji, L. Lanman

**Affiliations:** 1 The USC Brain Health Observatory, University of Southern California, 91001, Los Angeles, CA, USA; 2 Alzheimer's Research UK, CB21 6AD, Cambridge, UK; 3 Alzheimer's Disease International, SE1 4PU, London, UK; 4 Alzheimer's Society U.K., EC3N 2AE, London, UK; 5 F. Hoffmann-La Roche, 4070, Basel, Switzerland; 6 Center for Improving Chronic Illness Care, USC Dornsife, 635 Downey Way, #505N, 90089, Los Angeles, CA, USA

**Keywords:** Disease-modifying treatment, preparedness, capacity, Alzheimer's disease, dementia, NHS, investment, England, health system

## Abstract

**Background:**

As disease-modifying Alzheimer's (AD) treatments are becoming available, concerns have been raised that even high-income countries lack the diagnostic capacity to accurately identify eligible patients in a timely manner.

**Objectives:**

We analyze how much NHS England would have to invest in capacity for AD specialists, biomarker testing with PET scans or CSF testing and MRI scans to reach G7 average levels and estimate the effect on wait times in the diagnostic process.

**Design:**

Desk research and expert interviews for cost and capacity data. Markov model to estimate wait times.

**Setting:**

NHS England.

**Measurements:**

AD specialists, and PET and MRI scanners per capita in G7 countries and wait times in England under different investment scenarios.

**Results:**

England has the lowest number of PET and MRI scanners and the second-lowest of AD specialists per capita among the G7 countries. An investment of GBP 14 billion over ten years would be needed to reach G7 average levels, of which 31%, 22%, 10%, 37% would be devoted to capacity for memory assessment services, PET scanning, CSF analysis, and MRI scanning, respectively. This investment would reduce estimated average wait times by around 87% between 2023 and 2032.

**Conclusions:**

The NHS England has large gaps in diagnostic capacity for AD. Without substantial investments, AD patients in England would experience substantial wait times and avoidable disease progression.

## Introduction

The first drug, which removes amyloid deposits from the brain - lecanemab, was recently approved by the FDA on the basis of a phase 3 trial that demonstrated its ability to slow the progression of early-stage Alzheimer's disease (AD) (1). Donanemab, another anti-amyloid treatment that is currently being studied, also recently reported positive results from a phase 3 trial (2). These positive outcomes have significant implications for science and policy. They confirm the long-debated amyloid hypothesis that accumulation of these toxic proteins contributes to the development of this disease and for the first time provides an option to treat it causally rather than deal with its inevitable consequences. However, making such treatments available will present a formidable challenge, even for high-income countries with sophisticated health systems, due to the complexity of the diagnostic process and a large prevalent patient population.

Amyloid-directed treatments for AD are most likely to be used in the early symptomatic stages (mild cognitive impairment (MCI) and mild dementia due to AD) to slow, and potentially prevent the progression to more severe disease stages. Determining treatment eligibility for these cases is considerably more complex than for other preventative treatments, such as vaccinations or cholesterol-lowering drugs. Patients, who will potentially be eligible, will be identified in primary care settings because of subjective memory complaints or abnormal findings on a routine cognitive exam, which is not consistently done today (3). They will be referred to a memory assessment service or neurology clinic for confirmatory neurocognitive testing and determination of the etiology, ideally after ruling out other reversible causes, like substance use, depression and vitamin B12 deficiency, and detecting possible structural etiologies, such as a past stroke. If early-stage cognitive decline is confirmed, patients will then have to undergo biomarker testing for the presence of amyloid pathology, via either positron emission tomography (PET) scan or based on cerebrospinal fluid (CSF) assessment. While blood-based tests for the AD pathology are becoming available, there are not yet validated for routine clinical use (4). Patients receiving these therapies will also require regular monitoring visits including magnetic resonance imaging (MRI) imaging for effect and safety monitoring.

As a recent audit of memory assessment services in England (5) has found, patients are rarely offered specialized diagnostic tests today, which means that diagnostic capacity will need to be expanded significantly to make disease-modifying AD treatments widely available. A 2019 study estimated that 654,436 people with MCI and 315,142 cases with mild dementia lived in the U.K. (6). As even manifest dementia is only diagnosed in less than two-thirds of expected cases by September 2022 in England (7), a much smaller proportion of these early-stage cases is probably diagnosed at this point, suggesting a large backlog and raising the question of the NHS' preparedness for such a treatment. Indeed, a recent publication found that only 36% of old age psychiatrists believed that their service could adapt to deliver disease-modifying treatments within a year (8).

While the U.K. has long provided political leadership, support and funding for Alzheimer's research, paving the way for such treatments, investment into the required health system infrastructure to deliver them has not kept pace. With an estimated 5.04 AD specialists (neurologists, old age psychiatrists and geriatricians, who are trained in memory care) per 100,000 population and 1.2 PET scanners per 1 million population, England has the second lowest and lowest, respectively, capacity among the G7 countries. A recent publication has shown that the resulting capacity constraints could lead to long and persistent wait times of over 120 months (9), which would likely result in potentially avoidable disease progression to the point that some patients may no longer benefit from treatment. Against this background, the objective of this study is to estimate how much NHS England would have to invest over a 10-year horizon to bring its AD diagnosis infrastructure up to the average of the other G7 countries and to project the resulting changes in wait times for a disease-modifying AD treatment.

## Methods

### Comparative capacity data

The analysis focuses on four critical components of the clinical pathway for determining eligibility for a potential Alzheimer's treatment: Capacity of AD specialists, capacity to conduct biomarker testing with PET scans and CSF analysis to determine the Alzheimer's pathology, and number of MRI scanners, as an MRI scan is part of the recommended evaluation process for treatment eligibility (10). We used published data and expert input to determine capacity in England relative to her peer G7 countries (Canada, France, Germany, Italy, Japan, and the U.S.).

### Investment needs

We conducted a focused literature search for sources for fixed and variable costs of increasing capacity of those four components to G7 average numbers. Information from the literature was augmented with interviews of 10 experts representing clinical services, hospital management, policy and research (three old age psychiatrists, two neurologists, one geriatrician, one health services researcher focusing on dementia care and three GPs). We estimated investment costs over a ten-year horizon, beginning in 2023 and spread out approximately equally over 10 years until 2032. The ten-year horizon was chosen based on the fact that the last U.K. dementia plan was for that timeframe (11). As published cost estimates came from difference years, all estimates were first inflated to 2023 GBP and then inflated by the conventional three percent annually in subsequent years. We analyzed two additional scenarios: A high investment scenario to achieve the current NHS target of 18 weeks waiting time for treatment (12) and a low investment scenario that would reduce the capacity gap to the average of the other G7 countries by half. We operationalized the high investment scenario as reducing wait times to less than 18 weeks on average over the 10-year model horizon, because scaling capacity to achieve the less than 18-week target for the initial surge in demand when the treatments become available first would lead to idle capacity in later years,

### Simulation model

**Figure 1:**
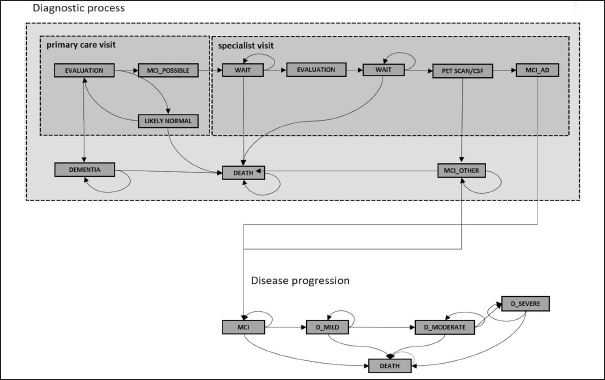
Schematic representation of the model

Our simulation model has previously been used to estimate expected wait times for a disease-modifying AD treatment in England (9), and the corresponding publication describes its full details and the model parameters (Appendix Table 1). In short, it simulates the journey of patients seeking evaluation for subjective memory complaints or as part of a wellness exam in primary care with two interacting layers. The first layer captures one of four true health states: cognitively normal, MCI due to AD, MCI due to other causes, and dementia using age and sex specific estimates for incidence and prevalence of MCI and dementia. The second layer captures a patient's journey through different evaluation stages: initial evaluation by a primary care clinician, neurocognitive testing by an AD specialist, confirmatory biomarker testing with PET scanning or CSF assessment and infusion delivery of the treatment. Specialist visits and biomarker testing are capacity constrained, and patients progress from cognitively normal to MCI and from MCI to dementia in the resulting wait times. Figure 1 shows a schematic representation of the model.

### Assumptions for patient journey

As the current absence of a disease-modifying treatment in England means that no data exist on the actual patient journey, we queried the above-mentioned ten experts for their professional opinion on how patients would likely progress through the different steps of their hypothetical journey, if a disease-modifying treatment were to become available. The below assumptions represent the average of their estimates:

• Starting in 2023, 25 percent of all individuals of age 50 and above, who have never been evaluated for cognitive decline, would undergo a brief cognitive test, like the Mini-Mental State Examination (MMSE), in primary care either because of a subjective memory complaint or as part of a routine assessment. Access to these visits is assumed to be unconstrained.
  • Five percent of individuals, who were previously found to be cognitively normal on a brief cognitive test, would return for another test in subsequent years.
Of the individuals found to be cognitively impaired, we assume that 80 percent would be referred to a dementia specialist, while 20 percent would be diagnosed with manifest dementia or cognitive impairment of reversible etiology, such as depression or alcohol use, and treated in primary care settings.
During the first AD specialist visit, neurocognitive testing would be performed and biomarker testing ordered for 90 percent of patients with confirmed MCI, having identified a different etiology in ten percent.
  • According to a survey by Alzheimer's Research U.K. (13), 42 percent of respondents stated they would be willing to undergo a lumbar puncture for CSF assessment, which is assumed not to be capacity-constrained, to confirm the Alzheimer's pathology. We assume that the remaining 58 percent of cases would require confirmation of pathology using a PET scan.
Of the expected 55.3 percent (14) with confirmed AD pathology, 80 percent would be determined to be eligible for treatment during a second specialist visit and undergo infusion treatment. The simulation prioritizes second specialist visits over first, i.e., appointments for first visits are only made available if no patient waits for his or her second visit.
Infusion delivery of the treatment is assumed to be not capacity constrained; the precedent of immunologic drugs in inflammatory diseases has shown that infusion capacity typically adjusts to new treatment options.

At each step, patients can be found to not have MCI due to AD based on test results, exit the queue for that year, and may reenter at the above-specified rate.

## Results

### Comparative capacity data

Table 1 shows the number of AD specialists, PET and MRI scanners for England and the remaining six G7 countries. Based on OECD data and a prior publication for England (15, 16), England has the lowest number of PET and MRI scanners, and, based on prior analyses (16), the second-lowest number of AD specialists per capita.

**Table 1:** Comparative capacity data for memory care infrastructure

	Specialists per 100,000 population	PET scanners per 1 million population	MRI scanners per 1 million population
Canada	4.94	1.52	10.06
France	6.46	2.48	15.38
Germany	24.02	1.63	34.47
Italy	15.58	3.55	30.22
Japan	11.08	4.70	55.21
USA	8.82	5.45	40.44
Average of other G7 countries	11.82	3.22	30.96
England	5.04	1.20	6.31
Capacity difference between England and average of other G7 countries	6.78	2.02	24.65


Source: (16, 17)

### Investment requirements and resulting increase in service availability

#### Alzheimer's disease specialists

We use the average annual consultant salary of GBP 166,744 as published in the NHS pay scale to reflect the cost of an additional Alzheimer's disease specialist and assume that each specialist could see 1,776 patients per year (Table 2). This assumption reflects an average of 1,332 clinical hours per consultant and year (17) and an estimated 45 minutes per consultation. Closing the gap to the G7 average would require adding 3,847 AD specialists over ten years for a total investment of around GBP 4.2 billion and provide approximately 38 million additional consultations (Table 3).

**Table 2:** Estimated fixed and variable cost for Alzheimer's disease care infrastructure

		Fixed cost	Cost per encounter		Source
Memory Assessment Service consultations			£	94	(12)
PET scanning	Device acquisition	£ 1,928,936	£	94	(13)
	Annual maintenance	£ 99,997	£	32	
	Software	£ 47,058	£	3	
	Building modification	£ 29,411	£	9	
	Tracer		£	1,059	
	Radiographer time		£	78	
	Radiologist time		£	41	
	Total		£	1,316	
CSF testing	Nurse training		£	150	(13)
	CSF analysis		£	235	
	Room utilization		£	29	
	Nurse time		£	244	
	Disposables		£	41	
	Total		£	700	
MRI scanning	Device acquisition	£ 1,890,166	£	25	(14)
	Annual maintenance	£ 100,809	£	17	
	Software	£ 90,350	£	3	(13)
	Building modification	£ 56,469	£	9	
	Radiographer time	£ 89,846	£	15	(14)
	Radiologist time	£ 185,400	£	31	(22)
	Total		£	100

**Table 3:** Overall investment cost over ten years for expanding England's Alzheimer's disease diagnostic infrastructure to G7 average levels and resulting expansion of services

	Fixed cost	Variable Cost	Total cost	Number of added services	Share of overall investment
Memory Assessment Services		£4,223,184,714	£ 4,223,184,714	37,573,973	31%
PET Scanners	£ 359,760,493	£2,700,700,149	£ 3,060,460,642	1,937,500	22%
CSF Analysis		£1,363,493,637	£ 1,363,493,637	1,627,500	10%
MRI Scanners	£ 3,279,790,957	£1,880,204,653	£ 5,159,995,610	46,194,000	37%
Total Investment			£13,807,134,603		

#### Biomarker testing capacity

Data for the cost of expanding biomarker testing capacity were derived from Wittenberg et al. (18), who had previously calculated the fixed cost of installing a PET scanner, including any necessary building modifications, the variable cost per scan and the cost per CSF analysis, including sample collection. Expressed in 2023 GBP, their estimates correspond to GBP 2,105,402 in fixed cost per installed scanner, GBP 1,316 per scan and GBP 700 per CSF test (Table 2).

Wittenberg et al. (18) estimated that a PET scanner can accommodate around 3,125 scans per year. Thus, the additional 97 devices to reach G7 average device density would provide capacity for around 1.9 million scans over ten years for a total investment of GBP 3,060,460,642.

As mentioned above, we assume that 42% of confirmatory biomarker tests would be based on CSF analysis (13), i.e., the number of CSF tests would increase proportionately to the to the additional PET scans that the investment would make possible. We estimate that an additional 1.6 million procedures would be performed over ten years for a total investment of GBP 1.4 billion (Table 3).

#### MRI scanners

According to a cost analysis from the Shrewsbury and Telford Hospital NHS Trust (19), fixed costs of installing an additional MRI scanner amount to GBP 1,890,166 for the device itself, GBP 56,469 for building modifications, and GBP 100,809 in annual maintenance in 2023 GBP. Their analysis did not account for the cost of a workstation and software for image processing and storage, and we are using the GBP 90,350 estimate for PET scanners (18) for a total fixed cost of GBP 2,137,795 per device. Since the average age at replacement is reported to be 12.8 years (20), no newly installed devices will need to be replaced during our 10-year timeline. Cost per scan is estimated as GBP 15 for the radiographer's and GBP 31 for the radiologist's time (Table 2).

We assume 20 scans per device each day, as 85% of facilities, which the Clinical Imaging Board surveyed for a 2017 study (21), scan between 10 and 30 patients per day, and brain scans without use of a contrast medium are relatively short. We assume scans are being conducted 300 days per year, since elective procedures are not likely to be performed on Sundays and Bank Holidays for a total of 6,000 scans per device annually. Around 46 million additional scans could be performed with those devices at a cost of approximately GBP 5.2 billion (Table 3).

#### Overall investment need to scale capacity to the G7 average

Table 3 summarizes the fully inflated investment requirements over the ten-year horizon from 2023 to 2032 for the NHS England to reach G7 average capacity as well as the additional services that those investments would secure. We assumed that the additional resources would be fully devoted to memory care. Overall investment over ten years would be around GBP 14 billion, of which 31% (GBP 4.2 billion) would be allocated to Memory Assessment Services, 22% (GBP3.1 billion) to PET scanning, 10% (GBP 1.4 billion) to CSF testing and 37% (GBP 5.2 billion) to MRI scanning.

#### Alternative investment scenarios

In order to achieve the NHS' target of 18-weeks average wait times, the investment would have to be close to GBP 16 billion, whereas closing half of the gap to the other G7 countries would reduce estimated investment needs to around GBP 10 billion. A detailed cost breakdown and the resulting increases in service volumes are shown in the Appendix Tables 2 and 3.

### Wait times projections

**Figure 2:**
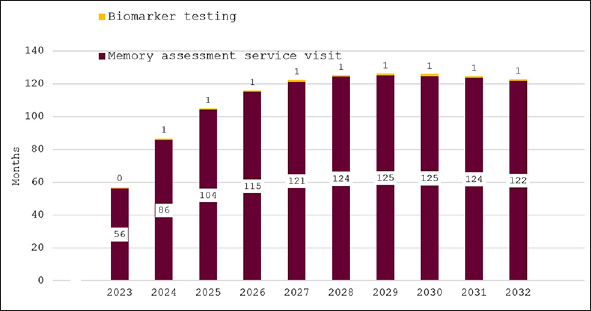
Wait times for determination of treatment eligibility for Alzheimer's disease (Months) - base case scenario

Figure 2 shows predicted wait times for our base case scenario of no additional investment in memory care. Average wait times for a specialist visit are estimated to be 56 months in 2023, increase to over 120 months by 2027 and remain at that level until 2032. Wait time for confirmatory biomarker testing is predicted to remain at around one month, because patients are held up in the long queue for an initial AD specialist appointment.

**Figure 3:**
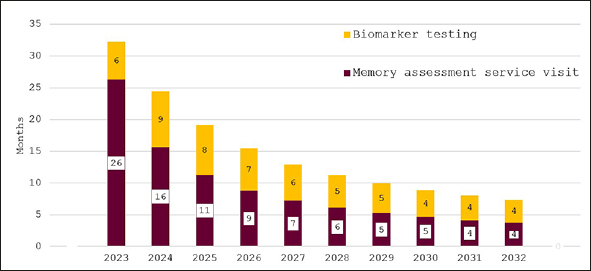
Wait times for determination of treatment eligibility for Alzheimer's disease (Months) - assuming investment to reach G7 average capacity levels

Figure 3 depicts the effect of bringing England's memory capacity up to the G7 average capacity over the ten years from 2023 to 2032. Wait times for an appointment with an AD specialist are projected to be around 11 months initially and then to decline quickly to around two months in 2032. Predicted wait times for confirmatory biomarker testing fall from three months in 2023 to around one month from 2029 on.

**Figure 4:**
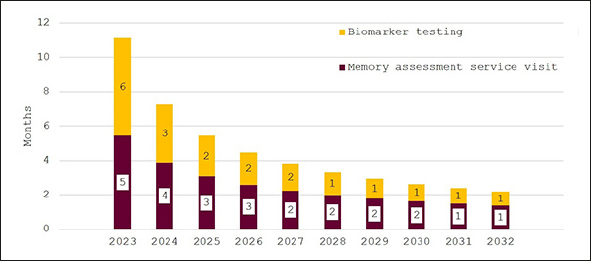
Wait times for determination of treatment eligibility for Alzheimer's disease (Months) - assuming investment to achieve target of 18-weeks average wait times

**Figure 5:**
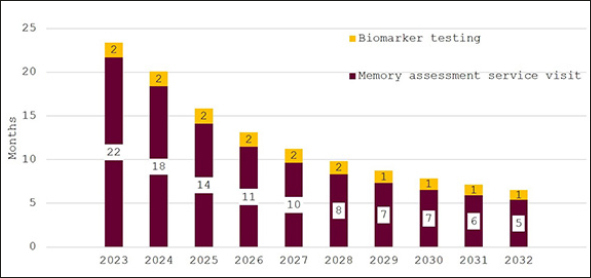
Wait times for determination of treatment eligibility for Alzheimer's disease (Months) - assuming investment to reduce gap to other G7 countries by 50%

In Figure 4, the effect of investing to achieve the NHS'18-week waiting time target is illustrated. While wait times are initially around eleven months, they decline quickly for an overall average wait of 18 weeks. Figure 5 shows wait times assuming that investments would only close half of the gap to the other G7 countries' averages with initial wait times of 24 months falling to around six months in 2032.

## Discussion

This study estimates the investment needed to increase NHS England's capacity to evaluate patients for the potential eligibility to receive a disease-modifying AD treatment to the average of the G7 countries as the base case scenario. In addition, we analyze a higher investment scenario to achieve the NHS' 18-week maximum wait time mandate and a lower investment scenario that would close the capacity gap to the other G7 countries by only half. The work builds on a prior study that had looked at the cost of expanding biomarker testing (18), expands the analysis to the full diagnostic pathway and also estimates the effect of investment on reducing wait times. Based on our literature search, this is the first such analysis performed for England or any other country or jurisdiction. We estimate for our base case scenario an overall investment need of around GBP 14 billion of which 31%, 22%, 10%, 37% would be devoted to capacity for memory assessment services, PET scanning, CSF analysis, and MRI scanning, respectively. The investment need would increase by around GBP 2 billion (14%) for the higher investment scenario and decrease by GBP 3.4 billion (25%) for the lower investment scenario. On an annual basis, these numbers amount to GBP 243, 279 and 183 per capita. The magnitude of the required investment amount reveals the consequences of a long-standing lack of investment in NHS infrastructure. According to OECD data, the U.K. together with Italy had the lowest capital expenditures as share of GDP among the G7 countries with an average of 0.4 percent between 2015 and 2019 (22). For comparison, capital expenditure was 1.1 percent for Germany and Japan, 0.7 percent for the U.S., 0.6 percent for France, which is the OECD average, and 0.5 percent for Canada. Of note, the additional PET and MRI scanners would have a useful life well beyond the 10-year horizon.

While there have been historic step changes in public spending on health services in the UK, this policy was driven by widespread recognition that service performance and health outcomes were dropping significantly in comparison to other European countries, such as in cancer care. The case for investment now in new treatments for AD comes at a time when the NHS England faces many challenges including surging patient demand and workforce recruitment and retention issues. However, the investment could be staged, emerging technologies could reduce investment needs, and novel financing arrangements, such as the National PET contract, under which companies install devices and receive payments per scan, could reduce upfront investment costs.

While it could be easy to dismiss the introduction of disease-modifying AD treatments as unaffordable, the profound impact of dementia across the health and care services must be acknowledged. As in cancer, the cost of delaying diagnosis and treatment for AD is high, as the loss of brain cells is irreversible. Between eight (23), and 14 percent (24) of patients with MCI progress to dementia each year and eventually to a dementia stage, for which a disease-modifying AD treatment may no longer be effective, and at which the disease will continue to progress to even more severe stages, associated with higher health and social costs. The cost of dementia was estimated to £32.1 billion in England in 2020 (18), when considering health care, formal and informal care costs. The implementation of disease modifying treatments offers the opportunity to slow down disease progression, reducing dependency on care and support and ultimately resulting in significant long term cost offsets.

### Limitations

This analysis has several limitations. We used a combination of published data and expert input to estimate average fixed and variable cost. Actual costs might differ from our estimates in either direction and vary substantially. In the absence of data on that variability we were unable to quantify the cost ranges. We accounted for cost of building modifications for MRI and PET scanners but not for new buildings and for room modifications for memory assessment services and lumbar punctures, which would add to investment cost. Non-financial obstacles, such as staff shortages, especially of specialists, and limitations of existing buildings to add modern imaging equipment, could impose limits on the absorptive capacity for investments, leading to slower capacity growth. Lower than expected demand of patients to be evaluated for an Alzheimer's treatment might reduce the need to expand diagnostic infrastructure. We acknowledge that our assumptions for transitions through the patient journey are based on expert opinion and could over- or understate actual demand for services. For example, if the NHS England restricted access to individuals matching the trial population, i.e., limited comorbidities and no co-existing pathologies that could partly account for cognitive decline as suggested in a recent review (25), the number of eligible patients would drop substantially. On the other hand, two drugs with different mechanisms of action and no ARIA risk are in phase 3 trials (26, 27), which - if approved - could broaden the eligible pool. Progress in diagnostic technology, such as blood and retinal tests for the AD pathology and digital cognitive assessments, are likely to become substitutes for existing technologies and reduce demand for and/or cost of diagnosis in the near future. However, we caution that those technologies are not likely to be available or approved in time for use in routine clinical practice or be able to replace PET or CSF for the confirmation of amyloid pathology prior to the launch of disease-modifying Alzheimer's treatments in England.

## Conclusion

The potential approval of the first of the disease-modifying AD treatments in the UK as early as 2024, and the prospect of subsequent availability in England, shines a light on the stark gap in diagnostic infrastructure needed to provide high-quality dementia care. While future detection and diagnostic technologies might allow for lowering investment levels in later years compared to our projections, the progressive nature of AD means that prolonged wait times would deprive numerous patients of the opportunity to receive a treatment while it would still be effective.

## Electronic Supplementary Material

Supplementary material, approximately 43.2 KB.

*Funding Source:* This research was funded through a research contract from F. Hoffmann-La Roche Ltd (Roche) to USC, but the sponsor had no role in the design of the study, the interpretation of the results, or decision to submit. Open access funding provided by SCELC, Statewide California Electronic Library Consortium.

*Conflicts of Interest:* Soeren Mattke serves on the board of directors of Senscio Systems, Inc., and the scientific advisory board of AiCure Technologies, Alzpath, and Boston Millennia Partners. He has received consulting fees from Biogen, C2N, Eisai, Genentech, Novartis and Roche/Genentech. Outside of the submitted work, USC has research contracts on which Dr. Mattke is PI with Biogen, Eisai and Roche/Genentech. Susan Mitchell is a paid employee of Alzheimer's Research UK. She has contributed to Advisory Boards for Biogen and Eli Lilly and been part of expert panels for Roche, with payment only for directly incurred travel and subsistence costs. Chris Lynch is a paid employee of Alzheimer's Disease International. Kirstie MacLean Kalonji is a paid employee of Alzheimer's Society UK. Lydia Lanman is a full-time employee of and stockholder in F. Hoffmann-La Roche. Zhehao Shi and Mark Hanson do not report any conflicts.

*Consent statement:* As the study did not constitute human subjects research per U.S. federal regulations (45 CFR 46, 102(f))20, it was exempt from IRB review, consent requirements and registration.

*Ethical standards:* As above, the study is not considered human subjects research.
