# Supplementary material for: Estimated Investment Need to Increase England's Capacity to Diagnose Eligibility for an Alzheimer's Treatment to G7 Average Capacity Levels
Source: J Prev Alzheimers Dis. 2024 Feb 7;11(4):1022–9. doi: 10.14283/jpad.2024.24 (PMC11266384; doi:10.14283/jpad.2024.24)
Supplement: Supplementary file 1 — Supplementary material, approximately 43.2 KB. [file mmc1.docx]

# Appendix

### Model parameters

Model parameters for the disease transition and diagnostic parts of the model and their sources are documented in Table 1. Parameters for sensitivity and specificity of the diagnostic tests and their sources were derived from prior publications ^1^ ^2^ ^3,4^. Parameters for population size and mortality were obtained from the Office for National Statistics^5^ ^6^, and for dementia prevalence from Alzheimer’s Research U.K. ^7^ Prevalence of MCI was calculated using the population data and age-specific prevalence estimates from a meta-analysis by Petersen et al. ^8^ and applied to the English population statistics. Transition probabilities from cognitively normal to MCI and MCI to dementia were obtained from meta-analyses by Gillis et. al ^9^ and Mitchell and Shiri-Feshki^10^, respectively.

## Appendix Table 1: Model parameters and sources

|  | Value | Reference |
| --- | --- | --- |
| Projected England population (50 years and older) | | |
| 2020 | 119,344,000 | ^5^ |
| 2025 | 126,287,000 |  |
| 2030 | 132,405,000 |  |
| 2035 | 138,792,000 |  |
| 2040 | 145,201,000 |  |
| 2045 | 151,636,000 |  |
| 2050 | 156,729,000 |  |
| Initial prevalence | | |
| Cognitively normal | 85% | ^8^ |
| MCI | 9% | ^8^ |
| Dementia | 6% | ^7^ |
| Proportion of MCI patients with Alzheimer’s disease | 55% | ^11^ |
| Annual mortality rate by age group (%) | | |
| 50-54 | 0.4 | ^6^ |
| 55-64 | 0.9 |  |
| 65-74 | 1.8 |  |
| 75-84 | 4.5 |  |
| 85+ | 13.6 |  |
| Hazard ratio for excess mortality | | |
| MCI | 1.43 | ^12^ |
| Dementia | 3.26 | ^13^ ^14^ |
| Annual transition probability | | |
| Cognitively normal to MCI, age 50-54 | 0.010 | ^15^ |
| Age 55-59 | 0.010 |  |
| Age 60-64 | 0.015 |  |
| Age 65-69 | 0.015 |  |
| Age 70-74 | 0.024 |  |
| Age 75-79 | 0.026 |  |
| Age 80-84 | 0.050 |  |
| Age >=85 | 0.074 |  |
| MCI to dementia | 0.065 | ^10^ |
| Initial and confirmatory tests | | |
| MMSE – Sensitivity | 0.82 | ^2^ |
| MMSE – Specificity | 0.73 |  |
| Blood-based biomarker test (Abeta42/40) – Sensitivity | 0.89 | ^1^ |
| Blood-based biomarker test (Abeta42/40) – Specificity | 0.69 |  |
| Confirmatory cognitive testing – Sensitivity | 0.95 | Assumption |
| Confirmatory cognitive testing – Specificity | 0.95 | Assumption |
| Confirmatory testing with CSF (pTau/Abeta42) – Sensitivity | 0.91 | ^4^ |
| Confirmatory testing with CSF (pTau/Abeta42) – Specificity | 0.89 |  |
| Confirmatory testing with PET – Sensitivity | 0.92 | ^3^ |
| Confirmatory testing with PET – Specificity | 0.95 |  |

## Appendix Table 2: Overall investment cost over ten years for expanding England’s Alzheimer’s disease diagnostic infrastructure to meet 18-week average wait times target and resulting expansion of services

|  | **Fixed cost** | **Variable Cost** | **Total cost** | **Number of added services** | **Share of overall investment** |
| --- | --- | --- | --- | --- | --- |
| **Memory Assessment Services** |  | £ 6,231,949,898 | £ 6,231,949,898 | 55,446,098 | 39% |
| **PET Scanners** | £ 359,760,493 | £ 2,700,700,149 | £ 3,060,460,642 | 1,937,500 | 19% |
| **CSF Analysis** |  | £ 1,363,493,637 | £ 1,363,493,637 | 1,627,500 | 9% |
| **MRI Scanners** | £ 3,279,790,957 | £ 1,880,204,653 | £ 5,159,995,610 | 46,194,000 | 33% |
| **Total Investment** |  |  | £ 15,815,899,787 |  |  |

## Appendix Table 3: Overall investment cost over ten years for expanding England’s Alzheimer’s disease diagnostic infrastructure to close half of the gap to G7 average levels and resulting expansion of services

|  | **Fixed cost** | **Variable Cost** | **Total cost** | **Number of added services** | **Share of overall investment** |
| --- | --- | --- | --- | --- | --- |
| **Memory Assessment Services** |  | £ 2,111,592,357 | £ 2,111,592,357 | 18,786,986 | 20% |
| **PET Scanners** | £ 251,010,964 | £ 1,914,379,877 | £ 2,165,390,841 | 1,375,000 | 21% |
| **CSF Analysis** |  | £ 966,506,697 | £ 966,506,697 | 1,155,000 | 9% |
| **MRI Scanners** | £ 3,279,790,957 | £ 1,880,204,653 | £ 5,159,995,610 | 46,194,000 | 50% |
| **Total Investment** |  |  | £10,403,485,505 |  |  |

# References

1. Palmqvist S, Janelidze S, Stomrud E, et al. Performance of Fully Automated Plasma Assays as Screening Tests for Alzheimer Disease-Related β-Amyloid Status. *JAMA Neurol*. Sep 1 2019;76(9):1060-1069. doi:10.1001/jamaneurol.2019.1632

2. Roalf DR, Moberg PJ, Xie SX, Wolk DA, Moelter ST, Arnold SE. Comparative accuracies of two common screening instruments for classification of Alzheimer's disease, mild cognitive impairment, and healthy aging. *Alzheimer's &amp; Dementia*. 2013;9(5):529-537. doi:10.1016/j.jalz.2012.10.001

3. Ovod V, Ramsey KN, Mawuenyega KG, et al. Amyloid β concentrations and stable isotope labeling kinetics of human plasma specific to central nervous system amyloidosis. *Alzheimer's &amp; Dementia*. 2017;13(8):841-849. doi:10.1016/j.jalz.2017.06.2266

4. Trzepacz PT, Hochstetler H, Wang S, Walker B, Saykin AJ. Relationship between the Montreal Cognitive Assessment and Mini-mental State Examination for assessment of mild cognitive impairment in older adults. *BMC Geriatrics*. 2015;15(1)doi:10.1186/s12877-015-0103-3

5. Office for National Statistics. National population projections: 2020-based interim. Accessed 07/25/2022, <https://www.ons.gov.uk/peoplepopulationandcommunity/populationandmigration/populationprojections/bulletins/nationalpopulationprojections/2020basedinterim>

6. Office for National Statistics. National life tables. Accessed 07/25/2022, <https://www.ons.gov.uk/peoplepopulationandcommunity/birthsdeathsandmarriages/lifeexpectancies/methodologies/nationallifetablesqmi>

7. Alzheimer's Research U.K. Dementia prevalence by age in the UK. Accessed 07/25/2022, <https://www.dementiastatistics.org/statistics/prevalence-by-age-in-the-uk/>

8. Petersen RC, Lopez O, Armstrong MJ, et al. Practice guideline update summary: Mild cognitive impairment. *Neurology*. 2018;90(3):126-135. doi:10.1212/wnl.0000000000004826

9. Gillis C, Mirzaei F, Potashman M, Ikram MA, Maserejian N. The incidence of mild cognitive impairment: A systematic review and data synthesis. *Alzheimers Dement (Amst)*. Dec 2019;11:248-256. doi:10.1016/j.dadm.2019.01.004

10. Mitchell AJ, Shiri-Feshki M. Rate of progression of mild cognitive impairment to dementia - meta-analysis of 41 robust inception cohort studies. *Acta Psychiatrica Scandinavica*. 2009;119(4):252-265. doi:10.1111/j.1600-0447.2008.01326.x

11. Rabinovici GD, Gatsonis C, Apgar C, et al. Association of Amyloid Positron Emission Tomography With Subsequent Change in Clinical Management Among Medicare Beneficiaries With Mild Cognitive Impairment or Dementia. *JAMA*. 2019;321(13):1286. doi:10.1001/jama.2019.2000

12. Vassilaki M, Cha RH, Aakre JA, et al. Mortality in mild cognitive impairment varies by subtype, sex, and lifestyle factors: the Mayo Clinic Study of Aging. *J Alzheimers Dis*. 2015;45(4):1237-45. doi:10.3233/JAD-143078

13. Neumann PJ, Araki SS, Arcelus A, et al. Measuring Alzheimer's disease progression with transition probabilities: estimates from CERAD. *Neurology*. Sep 25 2001;57(6):957-64. doi:10.1212/wnl.57.6.957

14. Aneshensel CS, Pearlin LI, Levy-Storms L, Schuler RH. The transition from home to nursing home mortality among people with dementia. *J Gerontol B Psychol Sci Soc Sci*. May 2000;55(3):S152-62. doi:10.1093/geronb/55.3.s152

15. Gillis C, Mirzaei F, Potashman M, Ikram MA, Maserejian N. The incidence of mild cognitive impairment: A systematic review and data synthesis. *Alzheimer's &amp; Dementia: Diagnosis, Assessment &amp; Disease Monitoring*. 2019;11(1):248-256. doi:10.1016/j.dadm.2019.01.004
